# Valuable Insights into the Immune Responses against Coronavirus Disease 2019

**DOI:** 10.31662/jmaj.2020-0095

**Published:** 2021-01-14

**Authors:** Toshiaki Kikuchi

**Affiliations:** 1 Department of Respiratory Medicine and Infectious Diseases, Niigata University Graduate School of Medical and Dental Sciences, Niigata, Japan

**Keywords:** Immune response, SARS-CoV-2, IL-6

In this issue of The JMA Journa, Tjan et al. reviewed the neutralizing antibody response against severe acute respiratory syndrome coronavirus 2 (SARS-CoV-2) and cytokine/chemokine release in patients with differing severities of coronavirus disease 2019 (COVID-19), by using their original data of 15 patients with COVID-19, in which 5, 3, and 7 patients were in asymptomatic, severe, and critical conditions, respectively ^(1)^. All patients were hospitalized at Hyogo Prefectural Kakogawa Medical Center, which is one of the 55 publicly designated medical institutions for infectious diseases in Japan. These data must be crucial to understanding the pathophysiology of COVID-19 and developing useful therapeutic, vaccination, or both strategies.

Functional neutralizing antibodies specific to SARS-CoV-2 are recognized as a crucial defense mechanism, resulting in viral neutralization and clearance ^(2)^. For this reason, Tjan et al. serially collected blood samples from each patient and performed a neutralization test against SARS-CoV-2 (Biken-2 strain) in a biosafety level 3 laboratory ^(1)^. Although many of the recent studies have used a pseudotyped virus for the neutralization assay, they sometimes draw inconsistent results with those using authentic viruses. Then, Tjan et al. analyzed the neutralizing activity of patients' serum samples by using authentic viruses. Their results indicated that the neutralizing antibodies from 10 patients with severe or critical COVID-19 had higher titers than those of asymptomatic patients. Consistent with these results, other previous reports also suggested that strong antibody responses might be associated with more severe COVID-19, and low antibody responses might be associated with higher rates of viral clearance ^(2)^. However, a recent study has shown that the presence of neutralizing antibodies is not associated with disease severity but that SARS-CoV-2-specific CD4^+^ T cells and CD8^+^ T cells are significantly associated with less severe COVID-19 ^(3)^. This result suggests that T cell-mediated immunity is more important in controlling SARS-CoV-2 infection than humoral immunity. In this context, the beneficial effect of on time convalescent plasma therapy to the clinical improvement of patients with severe and life-threatening COVID-19 was not demonstrated by open-label, multicenter, randomized clinical trials in Wuhan, China ^(4)^. The humoral immune responses during SARS-CoV-2 infection might be affected not only by clinical settings of diagnosing patients with COVID-19 but also by many other factors, such as age, race, ethnicity, sex, body mass index, and smoking status ^(2)^. A more comprehensive study will be needed to achieve conclusive proof of humoral immune responses during SARS-CoV-2 infection.

It is also suggested that cytokine release is associated with the severity of SARS-CoV-2 infection ^(2)^. Thus, Tjan et al. investigated the levels of cytokines, chemokines, and growth factors in the sera of patients with COVID-19 with different severities of the disease at various time points ^(1)^. In their study, clearly higher levels of cytokines and chemokines were observed mostly in patients with severe COVID-19 compared with those with milder COVID-19 at all time points. Especially, they found that the serum level of interleukin (IL)-6 was clearly increased in all patients with severe COVID-19 but was not increased in asymptomatic patients. IL-6 is secreted as an inflammatory cytokine from monocytes, macrophages, and dendritic cells activated by SARS-CoV-2 infection. The marked proinflammatory properties of IL-6 receive much attention for the pleiotropic functions in activating both the acquired (by B and T cells) and innate immune systems (by neutrophils, macrophages, and natural killer cells), which leads to cytokine release syndrome, acute respiratory distress syndrome, and then secondary hemophagocytic lymphohistiocytosis frequently observed in patients with severe COVID-19. Although the increased serum level of IL-6 in patients with severe COVID-19 agrees with the IL-6-centered scenario of COVID-19 exacerbation, IL-6 receptor blockade in moderately ill hospitalized patients with COVID-19 by using tocilizumab did not show favorable efficacy in preventing their intubation or death ^(5)^. The failure of tocilizumab to improve clinical outcomes now raises a clinical question of whether elevated serum level of IL-6 in patients with COVID-19 represents a mere host response to the infection of SARS-CoV-2, rather than the central role in the pathophysiology of hyperinflammatory syndromes associated with severe COVID-19.

The scientific information on immune responses to SARS-CoV-2 is updated daily, and the results are sometimes conflicting. The discordant results may be partly due to differences in the setting of the study (i.e., in acutely ill or convalescent subjects), definition of disease severity, and other factors. SARS-CoV-2 immunity remains to be further clarified to harness the COVID-19 outbreak.

## Article Information

### Conflicts of Interest

None

### Disclaimer

Toshiaki Kikuchi is one of the Editors of JMA Journal and on the journal's Editorial Staff. He was not involved in the editorial evaluation or decision to accept this article for publication at all.
